# Authoritarianism Beyond Disposition: A Literature Review of Research on Contextual Antecedents

**DOI:** 10.3389/fpsyg.2021.676093

**Published:** 2021-06-24

**Authors:** Caroline Schnelle, Dirk Baier, Andreas Hadjar, Klaus Boehnke

**Affiliations:** ^1^Department of Psychology and Methods, Jacobs University Bremen, Bremen, Germany; ^2^Institute of Delinquency and Crime Prevention, Zurich University of Applied Sciences, Winterthur, Switzerland; ^3^Department of Social Science, Institute of Education and Society, University of Luxembourg, Esch-sur-Alzette, Luxembourg; ^4^Department of Social Work, Social Policy and Global Development, University of Fribourg, Fribourg, Switzerland; ^5^Center for Sociocultural Research, National Research University Higher School of Economics, Moscow, Russia

**Keywords:** authoritarianism, personality, contextual, threat, socialization

## Abstract

A core debate in authoritarianism research relates to the stability of authoritarianism, i.e., whether it is a dispositional phenomenon socialized in early childhood or even genetically predisposed, or whether it is impacted by time-sensitive, exterior conditions. Whereas certain individual authoritarian tendencies emerge as a rather stable personality trait, there is also empirical evidence for a dynamic influence of external factors. This review article provides a conceptual multilevel framework for the study of authoritarianism and offers an insight into the state-of-research on socialization and situational influences, with a particular focus on threat. Findings are discussed with regard to key theories of authoritarianism.

## Introduction

Classical authoritarianism theory, as spelt out most prominently by Adorno et al. (1950), emphasizes the idea of authoritarianism as a stable personality trait that is not subject to sweeping changes throughout the life span. In that manner, a number of scholars have related authoritarianism to the Big Five personality traits. Specifically, lower levels of openness to experience (Akrami and Ekehammar, 2006; Stenner, 2009; Perry and Sibley, 2012; Hotchin and West, 2018) and higher levels of conscientiousness (Sibley and Duckitt, 2008; Dallago and Roccato, 2010; Nicol and De France, 2016) have been linked to authoritarianism. Asp et al. (2012) even offered empirical evidence for increased levels of authoritarianism in patients with damage to the ventromedial prefrontal cortex, thus strengthening the view of authoritarianism as a biologically influenced and ergo genetically determined trait.

However, at the same time, several prominent authors have unfolded a more dynamic perspective to the development of authoritarianism (e.g., Duckitt, 1989; Altemeyer, 1996; Feldman and Stenner, 1997; Oesterreich, 1999). Whereas individuals may exhibit more or less authoritarian tendencies rooted in their genetic disposition and early socialization experience, the current environment can and does influence its manifestation. Here, one has to distinguish between *situational* influences that are singular, time-sensitive events and *contextual* influences that shape the immediate life context in terms of the lifelong socialization experience and may vary throughout the life span. Obviously, this elicits the questions which individual and societal conditions exactly foster an “authoritarian reaction” and which factors shape this process. This review article aims to provide an overview of the situational and contextual factors that are to be distinguished when studying the impact of the external factors on authoritarianism. A theoretical framework will be outlined, allowing to identify situational and contextual influences on authoritarianism on three different levels: the macro-level (the society), the meso-level (institutions such as schools and peers), and the micro-level (individuals and their families).

The theoretical approaches will be backed by an overview of the existing literature. In order to provide an overview of concepts and empirical findings, we employed a specific methodology. Given the large amount of literature on authoritarianism—the database Scopus lists 2,063 scientific publications containing the term “authoritarianism” in the title (1900–2020), and the search engine Social Science Research Network links 747 publications to “authoritarianism” (1900–2020)—our main criterion in the selection of classical and contemporary publications was any indication of external influences on authoritarianism. That is to say, we selected conceptual and research publications that emphasized the situational and contextual nature of authoritarian orientations.

In the following section, we will outline the foundations of a contextual—rather than an essentialist, stable—perspective on authoritarianism and present a multilevel conceptual framework. Subsequently, we review theory and research regarding the mechanisms behind authoritarianism, presenting both the situational perspective, with a focus on threat, and the contextual perspective of lifelong socialization. In a subsequent section, the macro-level will be examined by reviewing the research on cultural antecedents. Finally, we briefly summarize the main outcomes of this review and draw conclusions for future research.

## Contextual Perspectives on Authoritarianism

What explanatory power does a context-sensitive perspective on authoritarianism add to the essentialist perspective viewing authoritarianism as a stable trait? A convincing indication for an interactive explanation of authoritarianism was found in a meta-analysis by Sibley and Duckitt (2008): In that study, the scores on an often-used right-wing authoritarianism scale (Altemeyer, 1996, p. 250) are “highly reactive to situational manipulations” and “to be changed by group socialization influences.” The authors argue that the right-wing authoritarianism scale rather measures social/ideological attitudes than stable personality traits or dispositions. They suggest authoritarianism is not stable across the life span but subject to contextual influence. When looking at contextual influence, one has to consider both critical life events, i.e., threat stimuli, like—currently—the SARS-CoV-2 pandemic, as situational influences and lifelong socialization processes as long-term contextual influences. The latter should be tied to the individual’s position in the life cycle, for which chronological age may serve as a proxy. Concerning situational threats, the central assumption is that critical life experiences lead to changes in the level of authoritarianism. These experiences often include threatening life events that urge the individual to seek compensation through adaptations in the attitudinal preference patterns. Mayer (1975) offered examples for such critical life events: the transition from childhood to adulthood (moving out of the parental home), changes in the family structure (marriage or divorce), and the transition into parenthood (birth a child). Furthermore, he described educational transitions, such as school-to-school transitions (e.g., upper secondary school to tertiary education), school-to-work transitions, or changes in status and class during the career. Similarly, other scholars have pointed to the influence of critical social experiences in the family, at school, at the work place, in clubs, in societies, and in the public sphere on values and attitudinal orientations (Mead, 1934; Schütz and Luckmann, 1979; Meier et al., 1983). Another important influence is the views of significant others, in the sense of subjective norms or normative beliefs (Ajzen, 1991). Albeit all these examples seem plausible, one can criticize that they often lack a clear distinction between the lifelong socialization process and a critical life event.

Recent research shows that authoritarianism undergoes change across the life span and can be manipulated experimentally (Pettigrew, 1999; Stellmacher and Petzel, 2005a). Even classical representatives of authoritarianism theory pointed out that contexts and conditions must be analyzed with regard to the development and the activation of authoritarianism: Adorno et al. (1950) rooted the authoritarian personality in situational factors, socialization practices, and the family environment/structure, suggesting that changes in social attachment and institutions directly affect authoritarianism. Later authoritarianism research has often distanced itself from the psychodynamic reasoning of classical authoritarianism theory. Instead of proposing psychodynamic “inner” explanations for the development of an authoritarian personality, Altemeyer (1988, 1996) proposed a conceptual framework derived from social learning theory, which emphasizes the influence of socializing agents, like the family or peers, and of the societal context.

To investigate the influencing factors of authoritarianism, an examination of its functions is crucial. Duckitt (2001) defined authoritarianism as a worldview closely linked to threat and fear, with a main antecedent in the frequent experience of punishment, in particular corporal punishment. Oesterreich (1996) yet more explicitly focused on the influence of lifelong socialization experiences and emotional processes on the internalization of authoritarian potentials, which are activated in times of crisis or rapid social change to deliver orientations for behavior. The function of the authoritarian mechanism is thus to compensate feelings of fear and insecurity. It provides an escape into a clear set of norms and regulations (Oesterreich, 1999; Hadjar, 2004). In line with the understanding of authoritarianism as put forward by Milgram (1974) or Bettelheim (1943), Oesterreich (1993) conceptualized authoritarianism as *both* a reactive human behavior in critical and fear-laden situations and a stable personality structure that prevents authoritarian individuals from seeking behavioral options other than the authoritarian reaction. Lederer (1995) speaks of a habitualized readiness to respond to situations of crisis by escaping toward institutions that provide security.

This approach called *the authoritarian dynamic* (Feldman and Stenner, 1997; Stenner, 2005, 2009) is based on previous findings on the relationship between threat and authoritarianism and the instability of authoritarianism over time (Stenner, 2005) as well as across environmental conditions (Sales, 1973; Doty et al., 1991). Accordingly, human beings carry different levels of an authoritarian predisposition, which activates the endorsement of authoritarian values and behaviors in the event of exterior threat stimuli (Stenner, 2005). This is not to say that a greater authoritarian predisposition activates a greater authoritarian reaction in face of threats. On the contrary, studies have shown a greater endorsement of authoritarian values after a perceived threat in individuals who have previously scored low on authoritarianism (Hetherington and Suhay, 2011; Mirisola et al., 2014; Norris, 2017; Linden et al., 2018; Carriere et al., 2019; Russo et al., 2020). Low-scoring authoritarians thus adjust their worldviews toward more authoritarian ways of thinking, whereas high-scoring authoritarians remain stable in their endorsement of authoritarianism. Mirisola et al. (2014) explain this adjustment with the loss of perceived control in low-scoring authoritarians, mediating the effect between threat and the increase in authoritarianism. Linking these findings to the Compensatory Control Theory (Kay et al., 2008, 2010; Landau et al., 2015) suggests authoritarianism to operate as an external source of control substituting a perceived lack of personal control. In support, Kay et al. (2008) found lower levels of personal control to be associated with higher support for governmental control. It is to conclude that in the event of threat, low-scoring authoritarians are more susceptible to a perceived loss of control, strengthening their authoritarian views as a compensatory mechanism.

As to high-scoring authoritarians, the question remains why they show elevated levels of authoritarianism even in the absence of threat. Previous research suggests that authoritarians are more inclined to have a more dangerous worldview (Altemeyer, 1988) and therefore live with a permanently elevated sensitivity toward threat (Cohrs and Ibler, 2009). Specifying these assumptions, Russo et al. (2020) delivered experimental evidence for an elevated anticipation of threat among high-scoring authoritarians. They described the findings as “a vicious circle whereby authoritarians tend to overestimate the societal threat they are exposed to, and this leads to a polarization of their initial attitudes and a greater endorsement of authoritarian political systems” (Russo et al., 2020, p. 94). High levels of authoritarianism may alleviate the negative effects of stressful life events on mental distress and thus serve as a coping mechanism (Van Hiel and De Clercq, 2009). In addition, Dunn (2017) suggests that authoritarians possess fewer cognitive skills helping them to cope with the stress caused by exterior threat.

**Figure 1:**
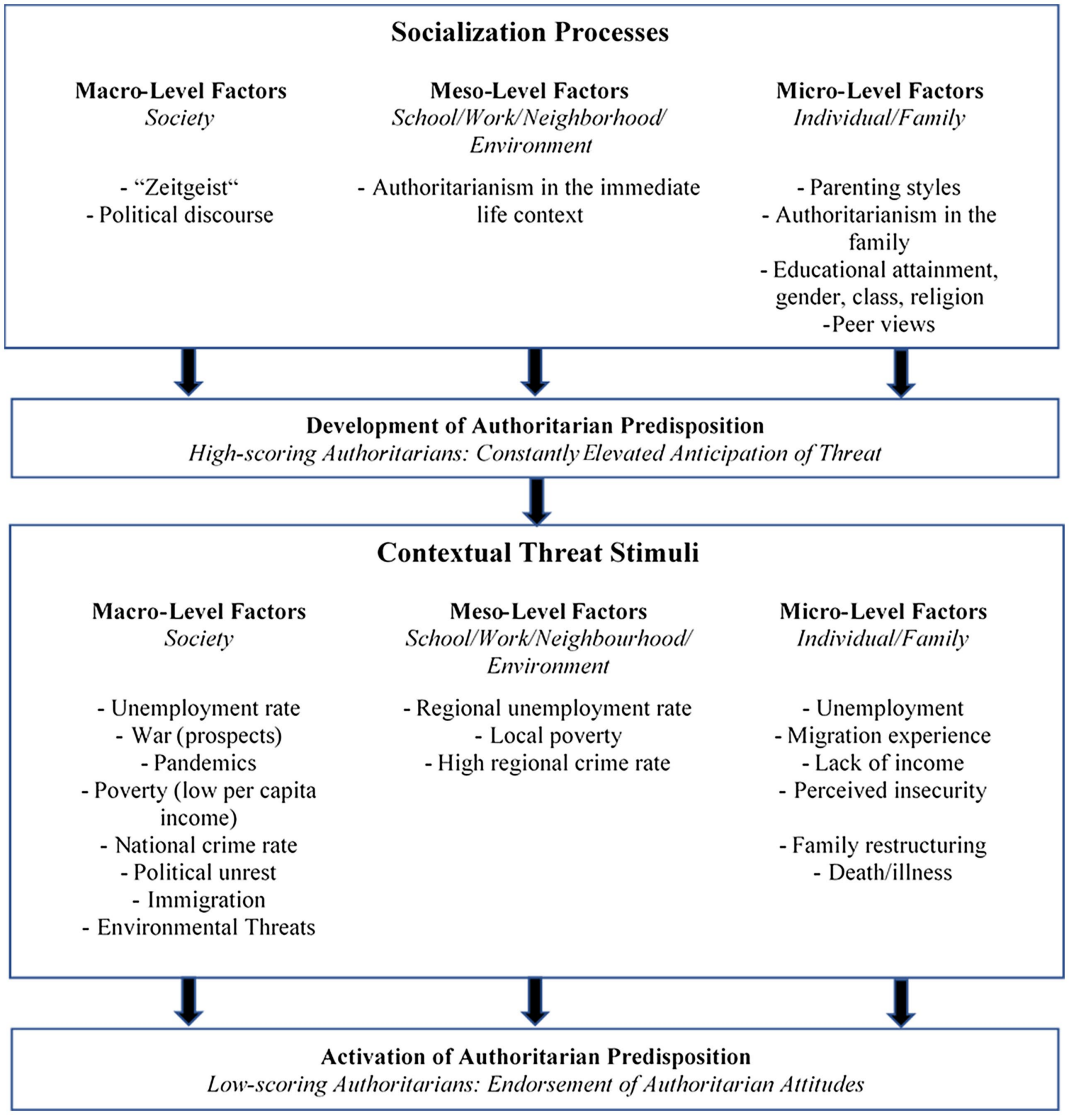
Multilevel model of the “Authoritarian Reaction” in high-scoring and low-scoring authoritarians.

Figure 1 shows an explanatory model, theoretically based on the model of group authoritarianism by Stellmacher and Petzel (2005b) as well as more recent findings by Mirisola et al. (2014) and Russo et al. (2020) on the adjustment of authoritarian worldviews in low-scoring authoritarians. It provides an overview of the “authoritarian reaction,” i.e., how contextual influences and lifelong socialization processes over three different levels impact authoritarian attitudes and behaviors among high-scoring authoritarians and low-scoring authoritarians.

In overall terms, two main features seem to be important when theorizing contextual factors of authoritarianism. They both play a distinctive role in the individual development and activation of authoritarianism. We will name this perspective as the *threat perspective* in terms of threats prompting an authoritarian reaction in a short-term process. Second, authoritarianism is subject to lifelong socialization experiences—and thus to long-term socialization processes—with a particular focus on socialization agents in the family, at school, in peer groups, or at the work place. This will be conceptualized as the *socialization perspective*. When investigating authoritarianism in an individual or a population, both perspectives have to be taken into consideration. In the next section, we present the state-of-research of each one.

### The (Multilevel) Threat Perspective: Theory and Findings

Combining the context-sensitive approach to authoritarianism, i.e., the *authoritarian reaction* (Oesterreich, 1993, 1996), and *integrated threat theory* (Sears, 1988; Quillian, 1995; Stephan and Stephan, 2000), appears highly fruitful when exploring situational mechanisms behind authoritarianism. From this perspective, authoritarianism unfolds as a reaction to rapid exterior change perceived as threat (Rippl et al., 2005). During such critical situations and times of insecurity, the individual (previously scoring low on authoritarianism) aims to compensate the feelings of threat by sticking to a simplistic authoritarian worldview, which provides rigid answers, clarity, and orientation. “Critical situations, such as those which require decision making, are the crucial points in a life course where opportunities for either the development of more individual autonomy or for the consolidation of a personality structure relying on authority arise. […] Situations that cause insecurity and anxiety are determined by social factors, such as society, social class, age, and gender” (Oesterreich, 2005, p. 283).

According to *integrated threat theory*, as proposed by Stephan and Stephan (2000), two categories of threat can be distinguished: *realistic threats*, which refer to the perception that a society (ingroup) is threatened in their economic wealth and/or physical safety, and *symbolic threats*, which are threats pertaining to the cultural structures in society, like norms, values, or symbolic systems (e.g., language). Although these dimensions can be separated empirically (Stephan et al., 1999, 2002), they are nevertheless interconnected: People who perceive high levels of *realistic threat* also tend to experience high levels of *symbolic threat*. Another taxonomy of threat has been proposed by Stephan and Renfro (2002) as well as by Rippl et al. (2007), who distinguish between *collective* and *individual* threats. Whereas a *collective threat* is a perceived threat regarding the entire society (e.g., increasing unemployment rates), an *individual threat* is based on the perception that personal wellbeing is in danger (e.g., individual unemployment). Furthermore, threats to social cohesion have been emphasized in the activation of an authoritarian reaction, as they disturb the desire for social conformity (Feldman and Stenner, 1997; Butler, 2013; Shaffer and Duckitt, 2013). Similarly, Stellmacher and Petzel (2005a,b) suggested a relationship between threat and authoritarianism in the case of a threat toward group identity.

Crowson et al. (2006, p. 737) offered two possible explanations for the close relationship between threat and authoritarianism: “(1) Perceived threat exerts a direct influence on the attitudes and beliefs of social perceivers, thereby leading them to demonstrate authoritarian attitudes and behaviors. (2) Perceived threat interacts with individual-difference characteristics […] to influence attitudes and behavior.” Feldman (2013) pointed out the difficulty of investigating these processes, as both may apply simultaneously. Low-scoring yet more so than high-scoring authoritarians may exhibit increased authoritarian attitudes that *both* react to perceived threats directly as well as in interaction with other threat-sensitive personal values like the desire for autonomy, social dominance orientation, or hierarchic self-interest (Baier and Hadjar, 2005).

Taking a closer look at the perceived threat stimuli, empirical studies focus on critical events not only on the individual but also on the societal level. When speculating about reasons for the increasing authoritarianism in the United States during the twentieth century, Lederer and Kindervater (1995) mentioned societal threats such as the Gulf War, the spread of HIV as well as a certain kind of a societal climate (“Zeitgeist”) summoning US citizens to be a proud American. Similarly, Stellmacher and Petzel (2005a) explored time periods characterized by a high threat level as a possible indication for increasing authoritarianism in the society, both regarding attitudes (e.g., high prejudice level) as well as behavior (e.g., voting behavior, conversion to authoritarian churches, anti-Semitic crimes). Specific indicators of high levels of threat on the societal level, or macro-level, are high unemployment rates, a low per capita income, a high inflation rate, an elevated frequency of “major crimes,” civil uprisings, strikes, and the country’s involvement in wars and other military action (Sales, 1972; Padgett and Jorgenson, 1982; McCann, 1999). Early 2000s research has paid special attention to threats regarding the physical safety after acts of terrorism (e.g., Norris, 2017). Initially, Crowson (2009) suggested a greater support of policies restricting civil liberties in the name of the war on terror among high-scoring authoritarians. However, Hetherington and Suhay (2011) found that under the influence of threat stimuli imposed by terror, the acceptance of restrictions is only increased among non-authoritarians.

A current example of threat on the societal level is the spread of SARS-CoV-2, a virus first identified in December 2019 in Wuhan, China, that by the first quarter of 2020, had spread across the globe (World Health Organization, 2020). The COVID-19 pandemic led many governments to introduce temporary authoritarian measures, such as restrictions on assembly and travel to mitigate the spread of the disease. Manson (2020) found that in face of the threat imposed by the virus, individual-level authoritarianism predicts the endorsement of such policies and practices translating to enhanced state control in the United States. Furthermore, Golec de Zavala et al. (2020) documented a rise in authoritarian attitudes during the outbreak of the pandemic beyond the regulations concerning COVID-19 in Poland: Here, the shift in authoritarianism spelled out in a desire for national cohesion followed by a rejection of dissenters to traditional sexual norms. Withal, studies relating macro-level threats and authoritarianism in the way just reported have to be treated with great caution, since the probability of an ecological fallacy—the drawing of false individual-level conclusions from macro-level findings (Robinson, 1950)—is rather high. What is largely lacking to this very day is studies looking at the link of macro-level threats and individual authoritarianism applying multilevel analyses.

Dealing with the influence of external threats on authoritarianism, social status may be considered as a safeguard, since a higher position provides better opportunities to deal with threats and class-specific socialization experiences can be assumed. In his working-class authoritarianism thesis, Lipset (1959) assumed that lower classes tend to have a more authoritarian view on politics and are thus more likely to support extremist movements blaming inferior-ranked scapegoats for problems and promising fast and simple solutions. According to Lipset (1959), authoritarian worldviews result from the fear of social relegation when belonging to a class of low socioeconomic status with a weak existential stability concerning job security and career opportunities. Enhancing factors may be low levels of education, the degree of isolation of the class, economic and psychological uncertainty, and the forms of family life prevalent in lower classes. This perspective was first criticized by Miller and Riessman (1961), stressing the existence of pro-democratic, left-wing-oriented groups among the working class and antidemocratic movements rising from the middle classes. Subsequent scholars emphasized the link between authoritarianism and lower levels of education, rather than the belonging to a lower social class (Lipsitz, 1965; Grabb, 1979; Dekker and Ester, 1987). The underlying idea is that education stabilizes one’s own economic status and reduces the susceptibility to simplistic, authoritarian prejudices against the outgroups.

Both the analyses of social status and of education—which are rather contextual than situational factors—as attenuating influences of the authoritarian reaction show that situational effects on authoritarianism cannot be studied without taking into consideration the socialization perspective. Critical life events are perceived, interpreted, and maneuvered divergently, depending on the immediate life context, whereas the immediate life context produces critical life events, and vice versa. That is to say that both perspectives must go hand-in-hand, rather than favoring one perspective over the other. The following section examines the key factors of the lifelong socialization process that influence the authoritarian reaction triggered by critical life events.

### The Socialization Perspective: Theory and Findings

Considering both individual causes of authoritarianism (micro-level) and factors of social authoritarianism (macro-level), an important socialization factor preventing high levels of authoritarianism is—as said—education. Several contemporary authors even argue that education is the decisive characteristic of non-authoritarians, as many studies exhibit robust findings on this link (e.g., Hopf, 1999, or Hadjar and Schlapbach, 2009 on postmaterialism as an antipole to authoritarianism). For example, Altemeyer (1988), Peterson and Lane (2001), and Peterson et al. (2016) showed that people who studied at institutions of tertiary education showed lower levels of authoritarianism than people who did not. In line with these findings are the results reported by Hadjar (2004), indicating that people with at least an upper secondary school degree exhibit lower levels of authoritarianism than their less educated agemates. Following the conceptual considerations of Hopf (1999) on the education-ethnocentrism link and the general concept on how education shapes world views by Hadjar and Becker (2009), education may influence authoritarianism *via* three distinct mechanisms: First, higher education results in greater cognitive skills and a cognitive mobilization that supports rather complex and tolerant non-authoritarian worldviews. As a result, highly educated people utilize fewer stereotypes (cognitive complexity assumption). Since higher-education institutions often include cooperation and perspective taking as themes of schooling, they also produce a higher degree of social competence (social competence assumption). Furthermore, highly educated individuals prefer postmaterialistic values as described in early work of Inglehart (1977) and are therefore less prone to exhibit ethnocentrism (value change assumption).

Second, education is linked to higher status, which provides better chances to produce subjective wellbeing and to cope with critical life events and with possible threats. In addition, the parenting styles, family structure, language style, work conditions, and ideologies among people from the higher classes exhibit less rigidity, likely originating from greater resource for coping with threats. Third, schools—as meso-level institutions linking society (macro-level) and individual (micro-level)—also function as socialization environments. In a hierarchically organized and highly stratified school system, as we find it in many European countries, higher-level schools and their teachers tend to support rather anti-authoritarian and open worldviews, leading cognitively mobilized students to confirm their non-authoritarian value systems. Highly educated individuals spend more time in educational institutions learning values and attitudes that are cherished by the ruling classes of a given society, such as low ethnocentrism (conformity assumption). However, as Mirshak (2020) points out, education can also be utilized as a hegemonic apparatus of authoritarian regimes to legitimize and protect their power, for example, by emphasizing certain knowledge, attitudes, and behaviors. This argument casts light upon the question whether authoritarianism levels are truly influenced by education per se—as a result of cognitive mobilization—or whether it is the political socialization experienced in educational institutions that leads to a higher or lower embracement of authoritarian values.

Another forming factor highlighted in the literature is the type of parenting style. Classic and modern authoritarianism studies suggest that parenting styles based on corporal punishment, a lack of warmth and emotional support by the parents, in combination with scarce participation rights for children, lead to higher levels of authoritarianism (Adorno et al., 1950; Hopf, 1993). Clemens et al. (2020) offered empirical support for this link but further emphasized that the relationship between authoritarianism and parenting style may also exist the other way around: Authoritarian individuals may be more likely to include corporal punishment in their parenting repertoire, resulting in a vicious cycle of authoritarian value transmission.

To assess the link between parenting style and authoritarianism, one has to examine the key characteristics of parental behavior. Based on Baumrind (1967, 1971) tripartite model of parenting styles (authoritarian, authoritative, and permissive), Maccoby and Martin (1983) introduced a two-dimensional framework, allowing to identify four different types of parenting styles. Mirroring the traditional dimensions of parenting—warmth and strictness (Sears et al., 1957)—they utilized the dimensions “responsiveness” and “demandingness” to describe authoritarian parents (demanding but not responsive), authoritative parents (responsive and demanding), indulgent parents (responsive but not demanding), and neglectful parents (neither responsive nor demanding). Following this quadripartite typology, a large body of research examined the effects of parenting styles on the offspring’s academic performance, psychosocial development, problem behavior, and conformity with norms (e.g., Lamborn et al., 1991; Steinberg et al., 1994). Traditionally, the authoritative parenting style (both responsive and demanding) was viewed as the optimal socialization agent for the development of children and youth (e.g., Baumrind, 1967, 1971; Lamborn et al., 1991; Steinberg et al., 1994). Authoritarian parenting (demanding but not responsive), on the other hand, was associated with a great variance in the children’s outcomes, pointing to acceptable academic performances and conformity with norms but lower levels of self-reliance and self-competence and higher levels of distress (Lamborn et al., 1991). A study focusing on parental support for autonomy as another aspect of parenting revealed that only individuals who received low parental support for autonomy responded to a societal threat situation with increased right-wing authoritarianism in an experiment (Manzi et al., 2017).

Studies looking beyond white, middle-class samples from Western societies revealed that the divergent impacts of authoritarian parenting on youth behavior result from contextual impacts, such as the ethnicity and socioeconomic status of the family (Quoss and Zhao, 1995; Park and Bauer, 2002; Dwairy et al., 2006). For example, authoritarian parenting reveals advantageous effects in ethnic minorities living in weak socioeconomic communities of the United States, offering protective benefits in hazardous contexts (Furstenberg et al., 1999). Furthermore, the effectiveness of parenting style highly depends on the cultural surroundings. In collectivistic cultures emphasizing discipline and harmony, an authoritarian family structure may offer the best preparation for future academic and work environments (Grusec et al., 1997).

Having said that the effectiveness of parenting styles largely depends on the cultural and social context, the parenting style itself may rather be regarded as a socialization context itself than a socialization practice (Darling and Steinberg, 1993). Garcia et al. (2019) argue that the optimal socialization style in today’s world changes with the current demands of a digitalized society. They suggest that a greater emphasis on responsiveness (i.e., the indulgent parenting style) may be beneficial, whereas the authoritarian parenting style may impose a risk factors for problem behaviors such as alcohol abuse (Garcia et al., 2020).

It is to note that parenting styles differ by the gender of the child, particularly in families that exhibit a patriarchal family structure and show a high level of traditional gender ideologies (Hadjar et al., 2007). These findings are based on the Power-Control Theory of Gender and Delinquency by Hagan et al. (1990). Gender is in fact one of the few demographic factors that receives wide attention in authoritarianism research. For instance, studies show that the links between authoritarianism and correlates are gender specific (e.g., interactions between gender and authoritarianism on career goals, educational aspirations, or marriage responsibilities, Peterson and Zurbriggen, 2010; interactions of threat and gender on dominance orientation, Sugiura et al., 2017). The same applies to transmission processes regarding right-wing extremist attitudes, with father-son transmission processes being stronger (Boehnke, 2017). Finally, twin studies even have indicated that the intergenerational transmission of authoritarian beliefs from the parents to the child may after all at least partly be rooted in genetic endowment (McCourt et al., 1999).

Although family is an important socialization agent, other socialization experiences must be considered, since their importance increases strongly after childhood. Altemeyer (1988), in particular, focused on the influence of a variety of interpersonal processes on authoritarianism throughout life, e.g., in schools, at the workplace, and during leisure time. Alongside the effect of parental socialization practices and mentalities in friendship networks (peers), personal experience with heterogeneity seems to have a powerful influence. Individuals who have frequently encountered people differing from them in characteristics like ethnic background, sexual orientation, socioeconomic status, political leaning, and/or religion tend to be less authoritarian, as they are less likely to think in black-and-white schemata and are more competent in dealing with otherness. This assumption corresponds to the intergroup contact theory (Allport, 1954; Pettigrew, 1998; Pettigrew et al., 2007), which postulates that both direct and indirect contacts with people of different ethnic origin, age, sexual orientation, or with handicaps, facilitates learning about outgroups, and supports the development of empathy and perspective-taking competencies. Both knowledge *about* and empathy *for* the outgroup members reduce the perception of threats and anxiety as well as the authoritarianism levels.

Social contact as a mechanism to reduce authoritarianism has been supported empirically: People with social ties to immigrants, even only having one ingroup friend who has an outgroup friend, showed less prejudice vis-à-vis people who differ from them (outgroup) and showed reduced levels of authoritarianism (Pettigrew, 1999; Pettigrew et al., 2007). In accordance with these findings, a study by Kalin and Berry (1980) showed that having undertaken extensive trips in one’s own country—in that case Canada—as well as to foreign countries goes along with lower levels of authoritarianism, as traveling seemingly fosters general flexibility and diminishes dogmatism and rigidity. As the study is correlational in nature, the finding suggests a socialization effect but could equally be interpreted as a finding that corroborates the impact of a personality trait, with low-authoritarianism individuals more prone to exhibit within-country and cross-border mobility. Yet, Stellmacher (2004) finding that only students who have left their place of upbringing experience a reduction in authoritarianism through education supports Kalin and Berry (1980) assertion that spatial mobility reduces authoritarianism.

Finally, a positive link between authoritarianism and individual attachment to groups was explored in a study by Duckitt (1989): If an individual shared the collectivist notion that ingroup goals are superordinate to personal goals, they showed a higher level of authoritarianism. This finding leads to the assumption that people who are integrated into a strong ingroup with a high sense of coherence, but also members of collectivist societies in general, exhibit higher authoritarianism. Building upon this idea, studies on social capital proposed that “bonding social capital,” i.e., ties between members of the same ingroup that have a sense of shared identity (Baron et al., 2000), is more prone to increase authoritarianism, whereas “bridging social capital,” i.e., ties between members of different ingroups (Baron et al., 2000), reduces authoritarianism. Since group coherence and outgroup contact are strongly shaped by the corresponding cultural context, these findings suggest to take a cultural perspective on authoritarianism. The following section will examine the theories and findings.

## Macro-Level Mechanisms: The Cultural Influence

While the mechanisms of threat and socialization relate primarily to individual and meso-level antecedents, culture appears to be an important (but under-researched) macro-level factor. Kornyeyeva and Boehnke (2013) argued for a re-inclusion of the original psychodynamic view on authoritarianism (Fromm, 1941). They were able to show that in autocratic societies (Russia and Turkey), but not in non-autocratic societies like Germany and other Western societies, the degree of—psychodynamically conceived—self-acceptance, as conceptualized by Berne (1964), explained individual-level authoritarianism above and beyond authoritarian parenting styles and an authoritarian societal climate.

Moreover, there are culture-specific macro-level socialization influences that may have an impact on the individuals’ authoritarianism level. Cross-cultural research has shown that neither the levels of authoritarianism nor its antecedents are universal, as an essentialist perspective might suggest. Based on data from 133 countries, Meloen (2000) showed that there is a strong relationship between culture, attitudes, and politics. State authoritarianism is strongly related to authoritarian attitudes among citizens that result from a culture that is based on hierarchies and traditional family structures. For instance, comparisons of authoritarianism among adolescents in East and West Germany after the fall of the Berlin Wall showed very clearly that different socialization cultures invoke different degrees of authoritarianism: East German adolescents agreed with authoritarian statements more than West Germans adolescents (Oesterreich, 1993). Boehnke and Rippl (1995) even showed that East Germans were more similar to US citizens than to West Germans concerning their levels of authoritarianism in the early 1990s. Further evidence on possible cultural causes of authoritarianism was provided by Zick and Henry (2009): Based on a large representative sample of Germans, they obtained that authoritarianism, in terms of an authoritarian reaction to particular life conditions and events, is higher among East (as opposed to West) Germans. Furthermore, less educated people, those with a lower income, and higher age showed a stronger authoritarian reaction (curvilinear effect). The same factors were highlighted in a study by Schmidt et al. (2006): Authoritarianism was shown to be decreasing with an increasing educational level and increasing by age. Women and, again, East Germans showed a higher authoritarianism level. Therefore, the interplay of cultural conditions and meso- and micro-level factors calls for further examination in future research.

## Conclusion

A main outcome of this review is the emerging impression that there are substantial conceptual backing and empirical evidence for contextual factors strongly affecting individual levels of authoritarianism. Both socialization factors and situational factors, such as threatening life events, have shown to be of significant influence on attitudes and behaviors in regard to authoritarianism. Current literature strongly suggests an interplay between the two; however, further research is needed to understand the underlying mechanisms. So far, empirical conceptualizations point to an increased anticipation of threats among high-scoring authoritarians (with strong authoritarian values mitigating the mental distress) and an enhancement of authoritarian values among low-scoring authoritarians in the occurrence of perceived threat (as a consequence of the perceived loss of personal control). It is still unclear whether a preexisting personality trait—possibly even genetically rooted in part—instigates the involvement in a specific socialization experience or whether it is the situational influence itself that enhances or impedes the authoritarian attitudes.

Another key question for future research should be whether situational factors merely activate the authoritarian reaction in low-scoring authoritarians at one specific point in time or whether they influence latent authoritarian predispositions over an extended period of time, e.g., by permanently increasing the anticipation of threat or need for group cohesiveness of an individual. In order to test that particular question cross-lagged panel analyses are needed, ideally based on a prospective long-term longitudinal study commencing in the childhood. Furthermore, we suggest for future studies to conduct multilevel analyses that allow an exact test of the impact of macro- and meso-level contextual factors on individual-level authoritarianism beyond a simple “social address” approach (Bronfenbrenner, 1986). A focus should be set on the interplay between higher-level influences and the individual reaction, as pursued in studies utilizing a quasi-experimental design, followed by analyses of variance as the statistical procedure of choice.

Seventy years after the publication of *The Authoritarian Personality* (Adorno et al., 1950), the interest in understanding and explaining authoritarianism is at an all-time high. While we have advanced from the idea of authoritarianism as a stable personality trait unaffected by external factors, there remains a large gap of knowledge regarding the interplay of situational and contextual influences.

## Author Contributions

DB, AH, and KB conducted a search for the literature until the publication year 2014, drafted a preliminary version of the paper, and sequentially edited the final version of the paper. CS conducted a supplementary literature search for the publication years 2015–2021 and revised the paper in accordance with that new search. All authors contributed to the article and approved the submitted version.

### Conflict of Interest

The authors declare that the research was conducted in the absence of any commercial or financial relationships that could be construed as a potential conflict of interest.
